# Phytochemical-rich foods inhibit the growth of pathogenic trichomonads

**DOI:** 10.1186/s12906-017-1967-x

**Published:** 2017-09-13

**Authors:** Sabrina M. Noritake, Jenny Liu, Sierra Kanetake, Carol E. Levin, Christina Tam, Luisa W. Cheng, Kirkwood M. Land, Mendel Friedman

**Affiliations:** 1 Department of Biological Sciences, University of the Pacific, Stockton, CA 95211 USA; 2 Healthy Processed Foods Research Unit, Agricultural Research Service, United States Department of Agriculture, Albany, CA 94710 USA; 3 Foodborne Toxin Detection and Prevention Research Unit, Agricultural Research Service, United States Department of Agriculture, Albany, CA 94710 USA

**Keywords:** *Trichomonas vaginalis*, *Tritrichomonas foetus*, Theaflavin, Flavonoid, Polyphenol

## Abstract

**Background:**

Plants produce secondary metabolites that often possess widespread bioactivity, and are then known as phytochemicals. We previously determined that several phytochemical-rich food-derived preparations were active against pathogenic foodborne bacteria. Trichomonads produce disease (trichomoniasis) in humans and in certain animals. Trichomonads are increasingly becoming resistant to conventional modes of treatment. It is of interest to test bioactive, natural compounds for efficacy against these pathogens.

**Methods:**

Using a cell assay, black tea, green tea, grape, pomegranate, and jujube extracts, as well as whole dried jujube were tested against three trichomonads: *Trichomonas vaginalis* strain G3 (found in humans)*, Tritrichomonas foetus* strain D1 (found in cattle)*, and Tritrichomonas foetus-*like organism strain C1 (found in cats). The most effective of the test substances was subsequently tested against two metronidazole-resistant *Trichomonas vaginalis* strains, and on normal mucosal flora.

**Results:**

Black tea extract inhibited all the tested trichomonads, but was most effective against the *T. vaginalis* organisms. Inhibition by black tea was correlated with the total and individual theaflavin content of the two tea extracts determined by HPLC. Metronidazole-resistant *Trichomonas vaginalis* strains were also inhibited by the black tea extract. The response of the organisms to the remaining preparations was variable and unique. We observed no effect of the black tea extract on common normal flora bacteria.

**Conclusions:**

The results suggest that the black tea, and to a lesser degree green tea, grape seed, and pomegranate extracts might present possible natural alternative therapeutic agents to treat *Trichomonas vaginalis* infections in humans and the related trichomonad infections in animals, without negatively affecting the normal flora.

## Background

Trichomoniasis, caused by the pathogenic trichomonad *Trichomonas vaginalis*, is one of the most common nonviral sexually transmitted infections in the world. It contributes to reproductive morbidity and facilitates transmission of the human immunodeficiency virus (HIV) [1]. Related trichomonads also cause disease in farm animals (cattle, pigs) [2, 3] as well as cats and dogs [4, 5]. Because some *T. vaginalis* strains have become resistant to the FDA-approved synthetic drug metronidazole, a need exists to develop alternative treatments, preferably based on safe natural products.

We previously reported that the tomato glycoalkaloid tomatine strongly inhibited the growth of the following three mucosal pathogenic protozoa strains that are reported to infect humans, cattle, and cats: *Trichomonas vaginalis* strain G3, *Tritrichomonas foetus* strain D1, and *Tritrichomonas foetus* strain C1, respectively [6]. The results suggest that natural food ingredients have the potential to prevent and treat trichomoniasis in animals and humans. It is of fundamental interest to determine if other edible plants, especially those that have been shown to inhibit the growth of pathogenic bacteria and viruses, also have the ability to inactivate pathogenic protozoa as did tomatine. We hypothesize that natural preparations which act by disrupting cell membranes resulting in cell death might also act by similar mechanisms against pathogenic protozoa. To offer a rationale for the present study, we will first briefly mention previously reported antibiotic activities of the natural preparations we will evaluate.

- Grape seeds, skins, and pomace, byproducts of wine production, are reported to exhibit antimicrobial, antifungal, and antiviral properties against foodborne, medical, and oral bacteria, microbial toxins, and parasitic protozoa (*Eimeria tenella*, *Trichomonas vaginalis*) [7–10]. These properties seem to be associated with their content of polyphenolic compounds.
- Jujube fruits and seeds from the plant *Ziziphus jujuba* contain many bioactive compounds that have been reported to exhibit numerous health-promoting properties, as well as antimicrobial activities against pathogenic bacteria (*Escherichia coli*) and fungi (*Candida albicans*) [11–13].
- Pomegranate fruit and seeds contain bioactive compounds. For example, a high-ellagic acid pomegranate preparation reduced the heat resistance of the virulent pathogen *E. coli* O104:H4 in ground chicken [14]. In vitro studies showed that pomegranate peel and seed extracts inactivated pathogenic bacteria and fungi [15, 16]. Wafa, et al. [17] found extracts of the peel to be highly antimicrobial toward *Salmonella enterica* serovars Kentucky and Enteritidis.
- Tea leaves produce catechins that seem to be involved in the defense of the plants against phytopathogenic insects, bacteria, fungi, and viruses. Fermentation of tea leaves, as in the production of black tea, stimulates the condensation of catechins into theaflavins. Tea extracts and individual tea compounds (catechins from green teas and theaflavins from black teas) are reported to exhibit anticarcinogenic [18], and antibacterial, antitoxin, antiviral, and antifungal properties in vitro and in food [19–23].

The objective of the present study was to determine using cell-based assays the potential of preparations from the above-mentioned edible plants to inactivate multiple strains of antibiotic-susceptible and antibiotic-resistant disease-causing pathogenic protozoa and to relate the content of pure theaflavins determined by HPLC to the observed inhibitory activities against the pathogenic protozoa.

## Methods

### Materials

Table 1 lists the commercial sources and catalogue numbers of the ten plant-based food-derived preparations evaluated in the present study. The synthetic drug metronidazole was from BD Diagnostics (Sparks, MD, USA), TYM Diamond media from Hardy Diagnostics (Santa Maria, CA, USA), *Trichomonas vaginalis* strain G3 from Patricia Johnson, University of California at Los Angeles, CA, USA, MSA1132, a clinical isolate which is cytotoxic in vitro and MSA1126, a clinical isolate that is highly resistant to metronidazole used to treat trichomoniasis, from W. Evan Secor at the US Centers of Disease Control and Prevention, Atlanta, GA, USA, *Tritrichomonas foetus* strain D1 from Lynette Corbeil, University of California at San Diego, School of Medicine, San Diego, CA, USA, and feline *Tritrichomonas foetus*-like organism (strain C1) from Stanley Marks, University of California at Davis, School of Veterinary Medicine, Davis, CA, USA.

**Table 1:** Sources of food-based commercial test products evaluated against pathogenic trichomonads

Name:	Components:
60% Catechin green tea extract	60% Catechins; Sigma (St. Louis, MO, USA) #P1204
80% Catechin green tea extract	98% Polyphenols:80% total catechins:50% EGCg; Swanson # NWF240
Low-theaflavin black tea extract	≥20% Theaflavins; Swanson #SWH218
High-theaflavin black tea extract	>80% Theaflavins; Sigma #T5550
Pomegranate fruit extract	~30% Punicalagins; Swanson #LE107
Pomegranate seed	70% Ellagic acid; Swanson #NEC009
Jujube fruit	Swanson #SW1166
Jujube seed	2% Triterpene saponins; Swanson #SWH092
Red wine grape extract	MegaNatural® Red Wine Grape Extract with Resveratrol:total phenolics 60%, anthocyanins 8%, trans-resveratrol 5%
Grape seed extract	90% Polyphenols; Swanson #SWH032

### Stock solutions of the test preparations

Plant powders (5% w/w) were solubilized in a solution of 1:1 autoclaved water to DMSO (density of DMSO approximated to 1 g/mL). The powders were first dissolved or suspended in DMSO. Then the water was added and the compound was resuspended. Solutions were stored in a freezer at −8 °C. Solutions were vortexed before use.

### Stock culture

Stock cultures of *T. vaginalis* strains were maintained in an 11-mL volume of TYM Diamond’s media (pH 6.2) at 37 °C for 24 h. Every 24-h cycle, 1000 μL of cells were passed into 10 mL of TYM media to maintain the culture. Clinical isolates MSA1126 and MSA1132 require anaerobic conditions for successful in vitro culture.

### Preliminary screenings

Preliminary inhibition screenings of the preparations were performed at 0.02% w/w on a consistent number of cells inoculated into 5 mL of completed TYM media. Two controls were carried-out, one without any additions (wild type parasites in TYM media only) and one containing parasites, TYM media, and DMSO at a final concentration of 0.1% wt. This amount of DMSO added was equivalent to the highest volume of DMSO used in the largest aliquot of stock solution. Preliminary screenings for MSA1126 and MSA1132 strains were carried out in an anaerobic chamber because these isolates cannot grow under standard trichomonad culturing conditions. The preliminary screens were incubated at 37.5 °C for 24 h before being counted using a hemocytometer. Percentage inhibitory activities were calculated by subtracting the amount of inhibition caused by the solvent (DMSO) alone from the cell counts of trials containing the different plant extracts (which were resuspended in DMSO). All preliminary screening trials were performed a minimum of three times to a standard error of ≤0.10.

### Inhibition concentration 50%

IC50 trials were performed by titration analysis at 0.0175% w/w, 0.015% w/w, 0.0125% w/w, and 0.01% w/w on a constant number of parasite cells inoculated into 5 mL of completed TYM media. The amount of DMSO added was equivalent to the highest volume of DMSO used in the largest aliquot of stock solution. As described above, both a wild type and a DMSO control culture were carried-out in parallel with the treated *T. vaginalis*. IC50 determination for MSA1126 and MSA1132 strains were completed in an anaerobic chamber since these isolates could not be cultured using traditional *T. vaginalis* methods. These tubes were left in a 37.5 °C incubator for 24 h before being counted. All IC50s were performed a minimum of three times to a standard error of ≤0.10. The software GraphPad Prism was used to calculate a theoretical value of the IC50 based on the data obtained from the titrations and constrained to zero. Except for cell counting, all procedures were performed in a biosafety cabinet.

### Disc diffusion screening of theaflavin-rich black tea extract on normal flora bacteria

Cultures of normal flora, non-pathogenic strains such as *Lactobacillus reuteri* (ATCC 23272, *Lactobacillus acidophilus* (ATCC 43560), and *Lactobacillus rhamnosus* (ATCC 53103) were grown in Lactobacilli MRS at 37 °C under anaerobic conditions. Stock solutions of compounds at 0.02% w/w, as well as vehicle control DMSO:H_2_O, were diluted to 100 μM in media and incubated with empty BDL-sensi-discs (6 mm) for 20 min at room temperature. Discs containing vehicle control, compounds, or various antibiotic discs [levofloxacin (5 μg), gentamicin (10 μg), and gentamicin (120 μg)] were placed onto the bacterial streaked agar plates and incubated overnight at 37 °C. Vehicle, compound, or antibiotic sensitivity was determined via measurement of zones of inhibition around each disc in mm.

### Analysis of theaflavin content

Samples were analyzed by HPLC under the following conditions: column, Discovery C18 (Supelco, Bellefonte, PA), 5 μm particles, 4.6 × 250 mm; eluent, 25% acetonitrile, 1 mM sodium citrate; UV absorption was monitored at 270 nm [24]. Chromatographic peaks were compared to authentic samples of theaflavin, isomeric theaflavin-3-gallate, and theaflavin-3,3-digallate obtained from Chromadex (Irvine, CA). Absorption characteristics of all three samples were similar. All the theaflavins are reported as theaflavin equivalents.

## Results

**Fig. 1:**
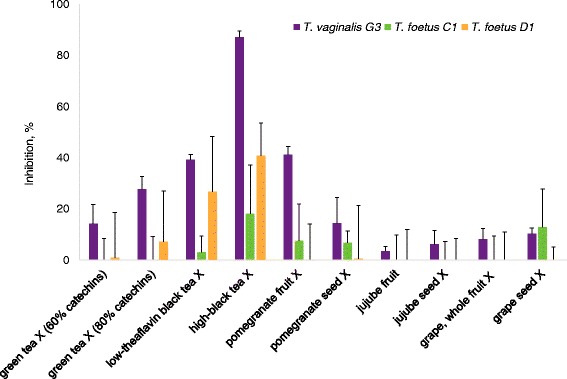
Inhibitory activity of ten plant preparations with the standard error (*n* = 3 or higher) on three different pathogenic trichomonads (*T. vaginalis* G3, *T. foetus* C1, and *T. foetus* D1). See Table 1 for sample sources. X denotes extract

Table 2 lists the average percentage inhibition by the ten test food-derived preparations of the different *T. vaginalis* strains under the described test conditions. To help visualize inhibition trends, the results are also presented as bar graphs shown in Fig. 1. It is interesting that only black tea extracts were effective against all three organisms whose response to the other compounds appears variable. It is also notable that of the three strains, G3 was most susceptible to inhibition by the natural products.

**Table 2:** Inhibition of parasite growth by natural plant preparations

Sample	% Inhibition		
	Human *T. vaginalis* (G3)	Feline *T. foetus* (C1)	Bovine *T. foetus* (D1)
60% Catechin green tea extract	14.2 ± 7.4^a^	0.0 ± 8.3^a^	1 ± 18^a^
80% Catechin green tea extract	27.7 ± 5.0	0.0 ± 9.2^a^	7 ± 20
Low-theaflavin black tea extract	39.3 ± 1.9	3.1 ± 6.3	27 ± 22
High-theaflavin black tea extract	87.1 ± 2.6	18 ± 19	41 ± 13
Pomegranate fruit extract	41.2 ± 3.2	8 ± 14	0 ± 14^b^
Pomegranate seed	14 ± 10^a^	6.8 ± 4.5	1 ± 21^a,b^
Jujube fruit	3.5 ± 1.7	0.0 ± 9.8^a^	0 ± 12^b^
Jujube seed	6.2 ± 5.3	0.0 ± 7.2^a^	0.0 ± 8.5^b^
Red wine grape extract	8.2 ± 4.0	0.0 ± 9.4^a^	0 ± 11^b^
Grape seed extract	10.2 ± 2.3	13 ± 15	0.0 ± 5.1^b^

Numbers within columns sharing a common superscript letter are not significantly different, *P* < 0.05

**Fig. 2:**
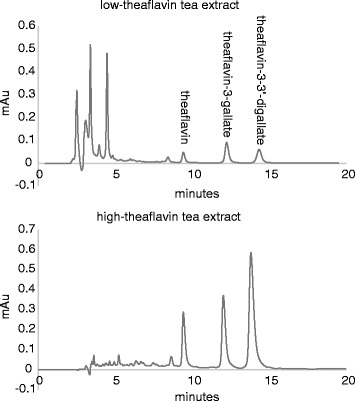
HPLC of theaflavin-containing black tea extracts. The equivalent of 20 μg of powder extract was injected onto the HPLC column for each chromatogram

Of the tested products, the black tea extracts were most effective. Figure 2 shows the HPLC chromatograms of the black tea extracts. Three of the theaflavins are well resolved, but the theaflavin-3-gallate and theaflavin-3′-gallate peaks co-elute, so these were reported as one. Table 3 lists the results of the HPLC analysis of the extracts. The low-theaflavin herbal extract was lower in theaflavins than the reported value on the label, while the high-theaflavin sample was considerably higher than the minimum value stated on the product specification.

**Table 3:** Theaflavin content of the two black tea extracts evaluated in the present study, determined by HPLC analysis

	Low-theaflavin black tea extract	High-theaflavin black tea extract
Theaflavin (TF)	2.30 ± 0.13	15.3 ± 1.0
Theaflavin-3-gallate (TF3G)	6.39 ± 0.18	25.2 ± 1.2
Theaflavin-3,3-digallate (TF33G)	5.35 ± 0.21	51.7 ± 2.5
Total theaflavins	14.04 ± 0.50	92.2 ± 4.7

*n* = 3 for low theaflavin black tea extract, *n* = 2 for high-theaflavin black tea extract. Listed values are in % (w/w)

**Fig. 3:**
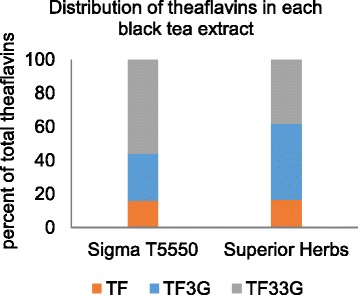
Relative amounts of the three theaflavins as a percent of total theaflavins for the two black tea extracts

Figure 3 shows the relative contribution of each of the individual theaflavins to the total theaflavin content. The low-theaflavin extract was relatively high in theaflavin-3-gallates, while the high-theaflavin extract contained high amounts of theaflavin-3,3′-digallate.

The inactivation of the pathogens by the two black tea extracts correlated with its content of theaflavins (Pearson correlation value of 0.89 for G3, 0.72 for C1, and 0.89 for D1, *p* < 0.05). There was no significant difference in the Pearson correlations among the different theaflavin dimers, suggesting that the different theaflavins act by a similar mechanism against the pathogenic protozoa. Although we could not calculate a numerical value, there also appears to be a correlation of green tea catechin content to inhibition in the G3 and D1 strains. The green tea extract had no effect on the C1 strain.

The data also show that the pomegranate fruit extract was about three times more effective (41%) in inhibiting the growth of the G3 strain than was the seed extract (14%). There was little difference between the two extracts for the C1 strain, and the D1 strain was not inhibited by either. Pomegranate fruit juices are high in anthocyanins, ellagitannins and other hydrolysable tannins, and phenolic acids [25]. The label on our pomegranate fruit extract claims a content of ~30% punicalagins, a type of ellagitannin. We assume the extract is also rich in the compounds mentioned above, but we do not know the distribution. Because we do not know the full composition of these extracts, we cannot further comment on the source of the activity.

The jujube extracts were only minimally effective and only in G3. The seed extract was more inhibitory than the fruit. Seeds contain more total phenolics and have higher antioxidative activity than the fruit [26]. Also, the composition of the flavonoids in the fruit and in the seed is quite distinct [26].

The red wine grape extract was only effective against G3, while the grape seed extract was effective against G3 and C1. In fact, for C1, the efficacy of seed extract was a close second to the high-theaflavin black tea extract. This is relevant because of the tested strains, C1 appears to be the most resistant to inhibition by the natural compounds. In relative terms, the C1 strain is especially sensitive to grapeseed. Understanding the reason for these different sensitivities to the grapeseed could lead to a better understanding of the organisms themselves. An analysis of the phenolic profile of different parts of 13 grapevine varieties (whole grapes, skin, seed, and pulp) showed that the most abundant phenolic compounds in the seeds were flavan-3-ols (gallocatechin gallate and catechin), similar to green tea.

**Fig. 4:**
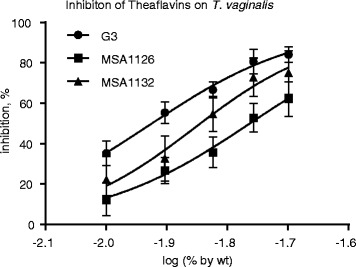
Dose-dependent response of theaflavin-rich black tea extract on three different *Trichomonas vaginalis* isolates; genome strain G3, metronidazole-resistant strain MSA1126, and cytoadherent clinical strain MSA1132

Black tea extract was the only preparation that inhibited all three trichomonads. To further explore the inhibition of the protozoa by black tea, the concentration-dependent inhibition of three *T. vaginalis* strains, G3, metronidazole-resistant MSA1126, and cytoadherent clinical strain MSA1132, by the theaflavin-rich black tea extract was determined, and IC50 values were calculated from the data. Figure 4 shows these dose-response plots. Inhibition of *T. vaginalis* G3 ranged from 23% to 86% for the tea extract concentration range of 0.01% by weight to 0.02% by weight. Using these titration data we calculated a theoretical IC50 value of 0.0118% w/w (R_2_ = 0.94). For strain MSA1126, the percent inhibition ranged from 12% to 62%, with a theoretical IC50 value of 0.0173% w/w (R_2_ = 0.88). For strain MSA1132, the percent inhibition ranged from 22% to 75%, with a theoretical IC50 value of 0.0140% w/w (R_2_ = 0.84). These theoretical value IC50 values were confirmed by direct testing.

Given that all of these pathogens reside in a mucosal environment with a complex microbiome of bacteria, we also screened theaflavin-rich black tea extract for activity against several common normal flora lactobacilli bacteria species. At the highest concentration of 0.02% by weight, no changes to growth of these bacteria were observed. This result suggests the possibility that these preparations may be effective without changing the normal microflora in the vagina in human trials.

## Discussion

As mentioned in the Background section, because all of the evaluated extracts were previously shown to inhibit the growth of foodborne pathogenic microorganisms, possibly by disruption of their cell membranes, our expectations that some of them might also inhibit *T. vaginalis* strains seem reasonable. Our results show that the theaflavin-rich black tea extract was the most effective against all the strains tested. Many of the other products were minimally effective.

Tea is a product of the plant, *Camellia sinensis*, which is high in flavan-3-ols, in particular, catechins. Both green and black teas originate from this same plant; the difference between them being in the processing technique. Green teas are processed quickly and by applying heat to the leaf to inactivate endogenous enzymes such a polyphenol oxidase, such that the catechins are retained in the final product. Black teas are left to enzymatically ‘ferment’ to produce oxidation products from the catechins. These products consist largely of dimers (theaflavins) and oligomers (thearubigins) [27]. It can be assumed that our black tea extract with low-theaflavin content, is likewise high in thearubigins. The results with these four tea extracts seem to suggest that the size of the molecule appears to be important. The smaller catechins and the larger thearubigins, although built from the same components, have lower efficacy against these pathogens.

The content of these compounds can vary significantly by sample [25, 28]. Ambigaipalan, et al. [29] compared free and bound phenolics in pomegranate fruit juices and seeds. The seeds were much higher in both phenolics and in other measures of antioxidative activity, with a significant portion of the activity due to bound phenolics. It would appear that protozoa are responding to some combination of bioactive compounds, rather than to total phenolic content.

Molecular dynamics simulations of the interaction of individual catechins and theaflavins with model lipid bilayers of cell membranes show that both classes of tea compounds (a) have a strong affinity with the artificial lipid bilayer; (b) are usually associated with cell membranes via hydrogen bonding to lipid headgroups; and (c) that some of the compounds are able to penetrate beneath the surface of the bilayer [30–32].

These events provide a foundation for understanding the mechanism of bioactivities of tea compounds and extracts. It is therefore likely that the black tea and green tea, and also possibly the pomegranate and grape fruit and seed extracts act by binding to and then disrupting protozoan cell membranes, resulting in cell death. It would be interesting to determine if the mechanism that operates against pathogenic bacteria is similar to that against pathogenic protozoa and whether plant essential oils and their components would also inhibit nonresistant and resistant protozoal strains.

Table 2 shows that of the three trichomonads, the human *T. vaginalis* G3 strain was most susceptible to inhibition by the plant preparations. The *T. foetus* C1 (feline) and D1 (bovine) strains had different sensitivities to the different preparations, with C1 being most resistant to inhibition. The inactivation of the pathogenic protozoa seems to be related to their individual and total theaflavin content of the tea extracts. In future studies it may be interesting to combine the different extracts to create broad-spectrum food-compatible antiprotozoal antibiotics. We do not know if individual phenolics present in these natural products may act antagonistically, additively, or synergistically in the cell assays. It would be interesting to test each of the pure compounds individually and in various combinations.

We previously surveyed the content of bioactive compounds in a variety of teas [21, 33, 34]. Our results showed that there are large differences in the content of bioactive compounds in widely consumed commercial black and green teas. In a follow up study, we found that storage of commercial green tea leaves for 6 months results in reduced total catechin content [35]. In addition, because preharvest environmental factors (soil fertility, climate, organic farming, and the use pesticides and biosynthetic stimulants) as well as postharvest events (processing, storage) are known to affect the nature and content of bioactive compounds in food plants, extracts from plants grown in different environments may not exhibit identical bioactivities. Taken together, these results suggest that it might not be possible to predict the efficacy of one extract from another against the protozoa.

## Conclusions

It seems that theaflavin-rich black tea extracts, and to a lesser extent catechin-rich green tea, pomegranate, and grape seed extracts, are effective under the described in vitro test conditions in inhibiting the growth of three parasitic trichomonads, and that the most potent of the preparations, the theaflavin-rich black tea extract, is effective as well against a metronidazole-resistant strain and a cytoadherent strain of *T. vaginalis*. On the basis of these results and our previous study on tomatine, there is a need for further clinical studies designed to evaluate both tomatine and black tea extracts to treat animal and human trichomoniasis using these food-compatible and safe products that would be expected to receive rapid approval for commercial sale by the Food and Drug Administration (FDA). Our studies on the inactivation of pathogenic protozoa by natural food-based preparations complement and extend previous reports by Land and colleagues on related studies on the inactivation disease-causing protozoa by synthetic compounds [36–39]. Finally, it is also relevant to note that a recent study by Setzer, et al. [40] using molecular modeling (docking) via computer simulation has the potential to predict anti-trichomoniasis properties of phytochemicals that act by a known pathway.
